# Development of measurement system for task oriented step tracking using laser range finder

**DOI:** 10.1186/1743-0003-10-47

**Published:** 2013-05-22

**Authors:** Tetsuya Matsumura, Toshiki Moriguchi, Minoru Yamada, Kazuki Uemura, Shu Nishiguchi, Tomoki Aoyama, Masaki Takahashi

**Affiliations:** 1 Graduate School of Science and Technology, Keio University, 3-14-1 Hiyoshi, Kohoku-ku, Yokohama, Kanagawa, 223-8522, Japan; 2 Research & Development Division, Murata Machinery, LTD, 136, Takeda-Mukaishiro-cho, Fushimi-ku, Kyoto, 612-8686, Japan; 3 Department of Physical Therapy, Human Health Sciences, Graduate School of Medicine, Kyoto University, 53 Kawahara-cho, Shogoin, Kyoto, Sakyo-ku, 606-8507, Japan; 4 Department of Physical Therapy, Graduate School of Medicine, Nagoya University, 65 Tsurumai-cho, Showa-ku, Nagoya, 466-8550, Japan; 5 Department of System Design Engineering, Keio University, 3-14-1 Hiyoshi, Kohoku-ku, Yokohama, Kanagawa, 223-8522, Japan

**Keywords:** Fall, Stepping performance, Measurement system, Laser range finder

## Abstract

**Background:**

Avoiding a fall requires fast and appropriate step responses, stepping speed as a fall risk indicator has only been assessed in older adults. We have developed a new measurement system that applies a laser range finder to assess temporal and spatial parameters of stepping performance such as step speed, length, and accuracy. This measurement system has higher portability, lower cost, and can analyze a larger number of temporal and spatial parameters than existing measurement systems. The aim of this study was to quantify the system for measuring reaction time and stride duration by compared to that obtained using a force platform.

**Methods:**

Ten healthy young adults performed steps in response to visual cues. The measurement system applied a laser range finder to measure the position and velocity of the center of each leg and of both legs.

We applied the developed measurement system to the rhythmic stepping exercise and measured reaction time and stride duration. In addition, the foot-off time and foot-contact time were quantified using the measurement system, and compared to the foot-off time and foot-contact time quantified using a force platform.

**Results:**

We confirmed that the measurement system can detect where a participant stood and measured reaction time and stride duration.

Remarkable consistency was observed in the test-retest reliability of the foot-off time and foot-contact time quantified by the measurement system (*p* < 0.001). The foot-off time and foot-contact time quantified by the measurement system were highly correlated with the foot-off time and foot-contact time quantified by the force platform (reaction time: *r* = 0.997, stride duration: *r* = 0.879; *p* < 0.001).

**Conclusions:**

The new measurement system provided a valid measure of temporal step parameters in young healthy adults.

The validity of the system to measure reaction time and stride duration was evaluated, and confirmed by applying to the rhythmic stepping exercise.

## Background

One-third of community-dwelling individuals, aged over 75 years experience at least one fall a year [1]. Falling occurs in various situations of daily life and generally results from an interaction of multiple and diverse risk factors [2,3]. Falling is a common problem in the growing elderly population and there is a need for effective and convenient fall-risk assessment tools that can be used in community-based fall prevention programs.

Avoiding a fall requires fast and appropriate step responses. Many previous articles have reported that reaction time can identify elderly individuals at risk of falling [4-8]. The step responses used to avoid a fall involve motor functions, cognitive functions, and visuospatial skills. In previous studies, reaction time, step length, and cognitive function including visuospatial skills and visuospatial working memory affected balance control and were important indicators of fall risk [9-15]. Effective fall risk assessment may therefore require the analysis of multiple parts of stepping performance such as stepping speed, reaction time, step length and step accuracy, rather than only one individual parameter. In this paper, step accuracy means whether the participant moved to the instructed target square.

Stepping performance is typically measured using a force platform, which is a useful tool with which to assess dynamic balance function and has good reliability [16]. However, this conventional measurement system is fixed and has been used for assessing only stepping speed as a fall risk indicator in elderly. In addition, the use of force platforms is inconvenient, expensive, and can measure only a limited number of parameters for stepping performance.

We have developed a new system for measuring step performance. The system uses a laser range finder (LRF) to assess the position and velocity of the center of each leg and of both legs, and uses this information to determine temporal and spatial parameters of the step including step speed, step length, and step accuracy. The LRF is smaller, less expensive, and more portable than force platforms, and the measurement system may therefore be useful for measuring stepping performance in a community-based fall prevention program.

The aim of this study was to investigate the test-retest reliability and validity of temporal and spatial parameters determined by this new measurement system during voluntary stepping in healthy adults.

## Methods

### Subjects

Students of Kyoto University were recruited as participants for this study. Ten men volunteered, none of who reported present or previous diseases or injuries associated with gait and/or balance impairments. Informed consent was obtained from all participants prior to participation, in accordance with the guidelines approved by the Kyoto University Graduate School of Medicine (approval number E-880) and the Declaration of Human Rights, Helsinki, 1975.

### Verification environment

**Figure 1:**
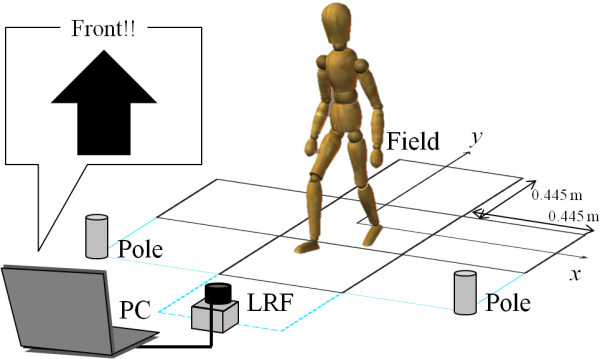
Measurement system for step tracking using a laser range finder.

Participants performed a rhythmic stepping exercise (RSE) [17]. The measurement system for the RSE is shown in Figure 1. At the beginning of the RSE, participants stood upright in the center of five squares (each 44.5 cm × 44.5 cm) arranged in a plus shape as shown in Figure 1. A visual cue was displayed on a monitor, and upon perceiving the visual cue participants were instructed to quickly move both legs into one of the four squares arranged around the starting position, and then return both legs to the center square. Performing this RSE under cognitive conditions may help improve dual-task ability, which simultaneously requires both cognitive functions such as reaction and short-term memory, and motor functions such as stepping in multiple directions. Stepping trajectory (position and velocity of each leg) and stepping performance (accuracy of step relative to the target square) were determined using the measurement system.

### System summary

The measurement system is manufactured by Murata Machinery Ltd. and consists of a LRF, a personal computer (PC), and two calibration poles. The scan period of the LRF is 0.1 s. The LRF can measure the distance within the error ±0.06 m in the stepping field. The center of the stepping field is calibrated by using the two poles. Thus, the environment required for the RSE is easily prepared and do not require the accurate installation of the LRF.

The system measured reaction time after the visual cue was displayed before the participant started to step as a cognitive parameter, and stride duration after the visual cue was displayed before the participant stopped to step as a temporal parameter. The system measured the positions and velocities of the center of each leg and of both legs and determined the accuracy with which the participant performed the task, i.e., stepped to the instructed target as spatial parameters. When a force platform is used to measure them, more than one force platform is needed because commonly-used force platforms do not cover the entire stepping field. However, by using the measurement system, we can measure movement parameters simply and inexpensively (the cost of LRF is about 1,000 dollars and the cost of a force platform is about 50,000 dollars). This system may be useful for measuring the stepping performance and can be used in community-based fall prevention programs.

The PC acquires data from the LRF, calculates the leg detection algorithm and displays a visual cue on a monitor.

**Figure 2:**
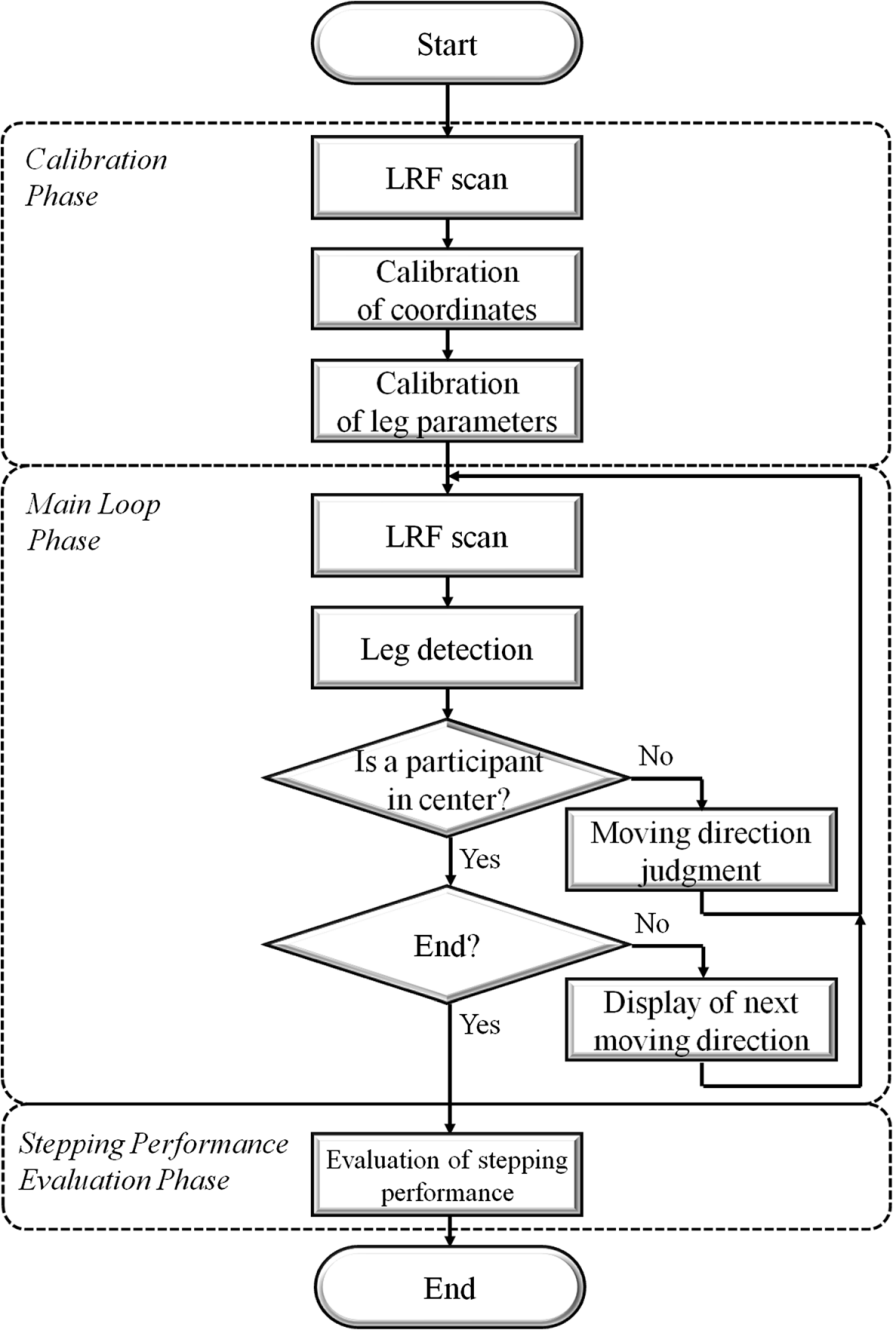
**The algorithm of the measurement system using laser range finder (LRF).**

The process followed by the measurement system is shown in Figure 2, and is divided into three parts: calibration phase, main loop phase and stepping performance evaluation phase.

### Calibration phase

In the calibration phase, the coordinate system for the stepping field was configured and the parameters required for the leg detection algorithm were identified.

**Figure 3:**
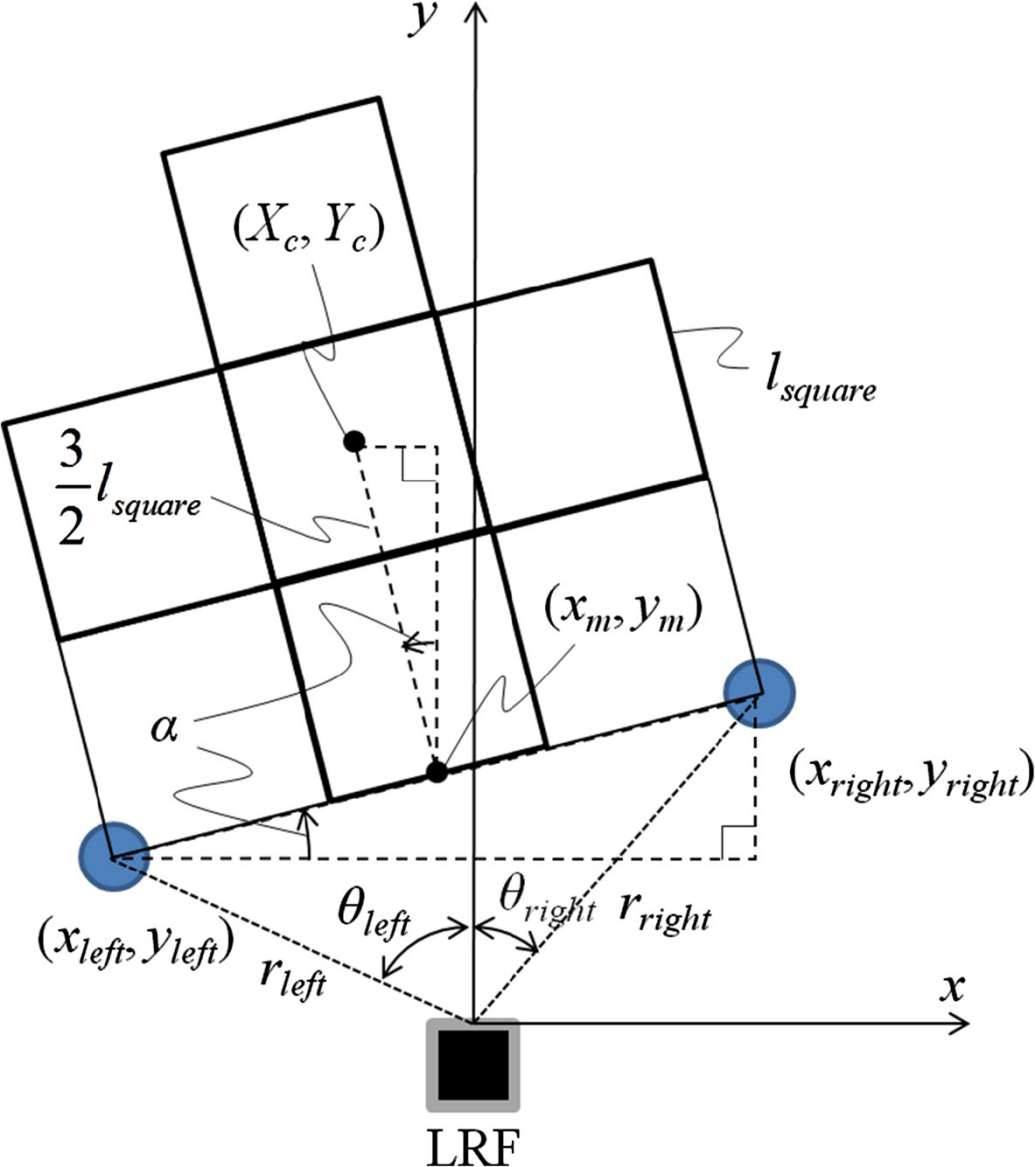
Calculation of the angle and the coordinate of the center of the stepping field.

The coordinate system for the stepping field was configured based on the two calibration poles that were symmetrically placed at the left- and right front corners of the stepping field as shown in Figure 1. The position of the poles was detected by the LRF at the beginning of the RSE and used to calculate the center and angle of the stepping field by using distance and angle to the poles obtained from the LRF. Even though the stepping field was not at the front of the LRF, the coordinate system was converted to the stepping field coordinate system as shown in Figure 3.

The coordinates of the poles, (*x*_*right*_, *y*_*right*_) and (*x*_*left*_, *y*_*left*_), was determined from distance and angle data obtained from the LRF. As shown in Figure 3, the angle of the field *α* was calculated as follows:

(1) $$\alpha =\arctan\left(\frac{y_{\mathrm{right}}-y_{\mathrm{left}}}{x_{\mathrm{right}}-x_{\mathrm{left}}}\right)$$

The coordinate of center of the poles (*x*_*m*_, *y*_*m*_) was calculated as follows:

(2) $$\left(x_{m},y_{m}\right)=\left(\frac{x_{\mathrm{right}}+x_{\mathrm{left}}}{2},\frac{y_{\mathrm{right}}+y_{\mathrm{left}}}{2}\right)$$

The coordinate of the center of the field (*X*_*c*_, *Y*_*c*_) was calculated as follows:

(3) $$\left(X_{c},Y_{c}\right)=\left(x_{m}-\frac{3}{2}l_{\mathrm{square}}\sin\alpha ,y_{m}-\frac{3}{2}l_{\mathrm{square}}\cos\alpha \right)$$

Where, *l*_*square*_ is the length of one square. Thus, the environment required for the RSE was easily prepared and did not require the accurate installation of the LRF.

**Figure 4:**
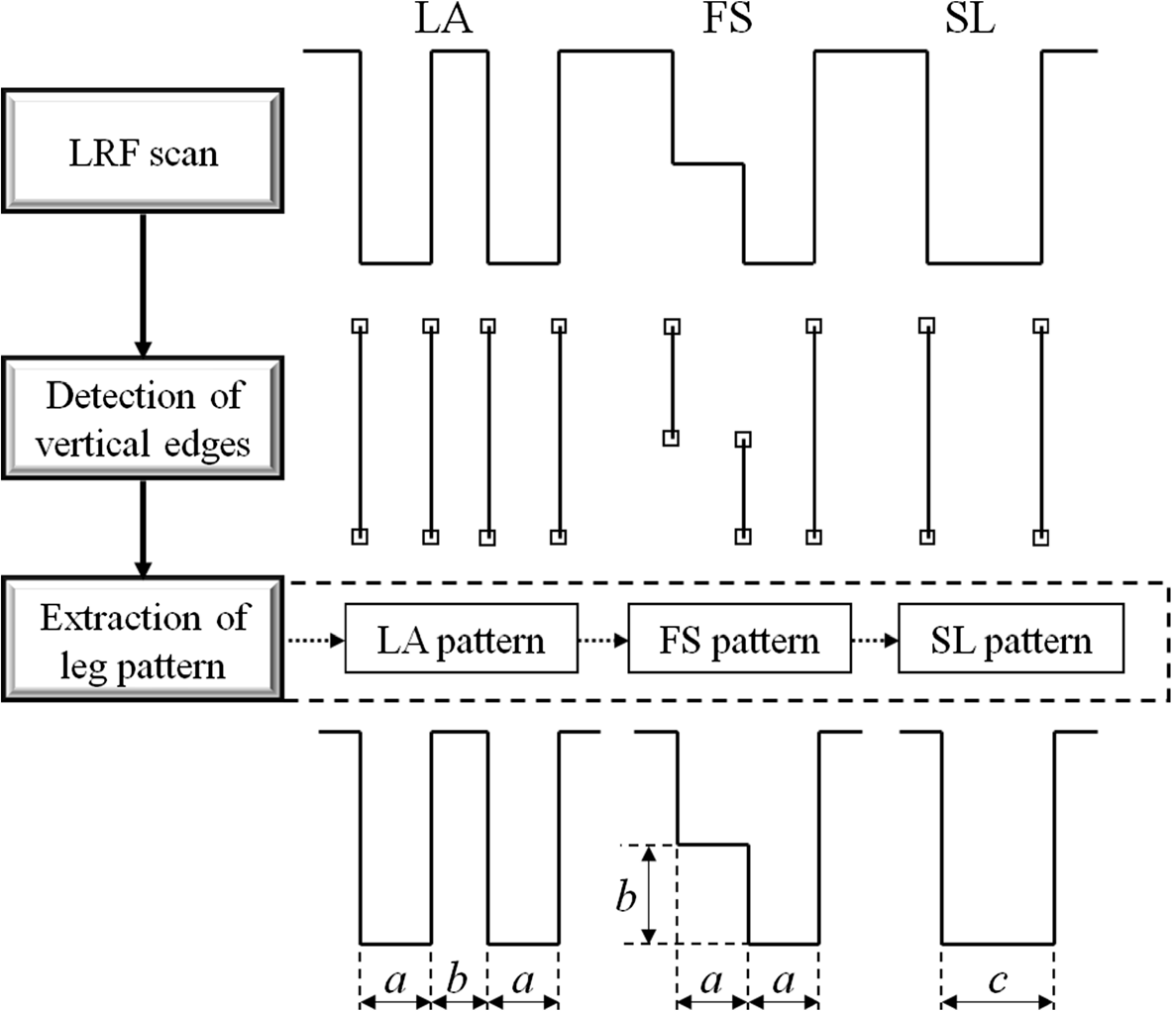
**Schematic representation of the leg detection algorithm **[18]**.**

As shown in Figure 4, the width of each participant’s leg was identified as parameters (*a*, *b* and *c*) used in the leg detection algorithm [18]. The legs of the participant who stood upright in the center were detected by the LRF before the start of the RSE, which was performed simultaneously with the configuration of the coordinate system fixed for the stepping field. The LRF used distance and angle data to determine the width of each leg and the parameter values were determined from the scan data on average ten times. RSE could then be performed without considering the differences in leg width of each participant. This allowed for the accurate measuring of step performance even if a large number of participants were targeted.

### Main loop phase

The main loop phase consisted of three parts: leg detection by LRF data, the judgement of whether the participant moved to the target square and the display of the next visual cue if the participant stood upright in the center.

Leg detection by the LRF data (leg coordinate data and the center-of-leg coordinates) was determined from distance and angle data obtained from the LRF scan, and was used in the leg detection algorithm proposed by Bellotto [18]. The leg detection algorithm in [18] measures only the position of the center of the legs; however, the leg detection algorithm in the measurement system measures not only the position of the center of the legs but also the positions of both legs. This is expected to improve the accuracy of the step performance measurements.

The leg detection algorithm is shown in Figure 4. We classified the legs into three leg patterns: LA (two Legs Apart), FS (Forward Straddle) and SL (Single Leg). In classifying the legs into the three leg patterns, we define *a*, *b* and *c*, which are, respectively, the leg width, the maximum step length and the width of the two legs together. These were identified in the calibration phase. First, we scanned a two-dimensional surface with the LRF. Next, the vertical edges were extracted from LRF data. Then, the leg pattern was detected by using three leg parameters and the combination of the arrangement of the edges. Finally, we calculated the position and velocity of the center of each leg and of both legs together using LRF data.

The square on which the participant moved to was determined using the position of the center of each leg and of both legs calculated in the leg detection algorithm and the coordinate system fixed for the stepping field. If the legs of the participant were inside the target square, the symbol “Ο” was displayed. On the other hand, if the leg was outside the target square, the symbol “Δ” was displayed, or if both legs were outside the target square, the symbol “×” was displayed.

We evaluated whether the participant moved to the exact target, and displayed the next visual cue when the participant returned and stood upright in the center of the stepping field.

### Stepping performance evaluation phase

**Figure 5:**
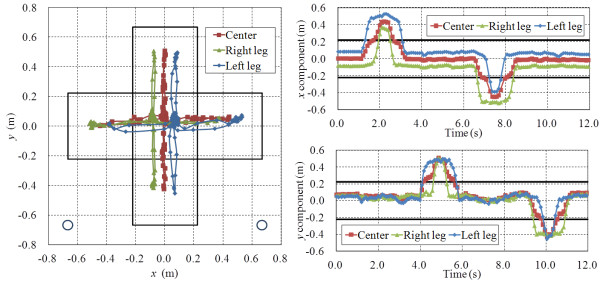
Trajectory of the center of each leg and of both legs.

**Figure 6:**
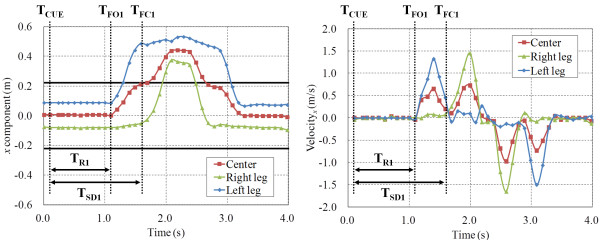
**Trajectory and velocity of *x *between 0.0 s and 4.0 s.**

A stepping trajectory of the center of each leg and of both legs from a single participant is shown in Figure 5. Figure 6 shows the *x*-directional position and velocity between 0.0 and 4.0 s.

The stepping trajectory obtained by the leg detection algorithm is shown in Figure 5. We measured foot-off time and foot-contact time from the position and velocity shown in Figure 6. The foot-off time of the leading leg (T_FO1_) was defined as the point at which the velocity started to increase. The foot-contact time of the leading leg (T_FC1_) was defined as the point at which the velocity is less than the threshold. In this paper, the threshold is 0.25 m/s. The reaction time of the leading leg (T_R1_) was calculated from the time that visual cue was displayed (T_CUE_) and T_FO1_. Stride duration of the leading leg (T_SD1_) was calculated from T_FO1_ and T_FC1_. Stride length was calculated from the positions at T_FO1_ and T_FC1_.

Thus, in the stepping performance evaluation phase, the velocity was calculated from the positional data of the center of each leg and of both legs after RSE ended. T_R1_, T_SD1_ and stride length were calculated from the velocity and displayed immediately on the PC.

### Test 1: application to the rhythmic stepping exercise

In order to verify the developed measurement system described above, we applied the system to the RSE and measured the stepping trajectories and stepping performance. Ten participants stepped forward, backward, and sideways (a total of 4 steps), and we measured their steps.

### Test 2: statistical analysis of the test using a force platform and the measurement system simultaneously

In order to verify the efficacy of T_R1_ and T_SD1_ that were obtained by the developed measurement system, T_R1_ and T_SD1_were measured by using a force platform and the system simultaneously. The force platform was a portable Kistler 9286 Force Platform. In case of the force platform, T_R1_ and T_SD1_ were calculated according to methods of Melzer et al. [16]. Ten participants stepped forward after the visual cue was displayed four times.

The test-retest reliability of T_R1_ and T_SD1_ determined by the system was assessed using the intraclass correlation coefficients ICC_1, 1_ with T_R1_ and T_SD1_ calculated using the force platform data. The criterion-related validity was determined using the Spearman’s correlation coefficient between T_R1_ and T_SD1_ obtained by the measurement system and T_R1_ and T_SD1_ obtained from the force platform. Data were analyzed using the Statistical Package for the Social Sciences (Windows version 19.0).

## Results

### Test 1: application to the rhythmic stepping exercise

**Figure 7:**
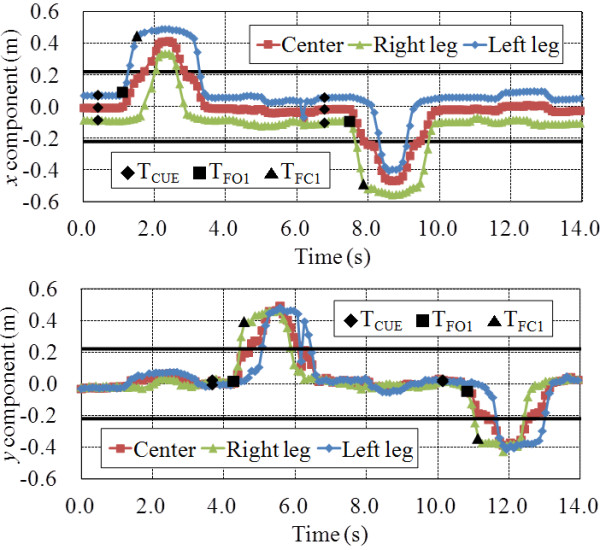
Results of step tracking.

The results of one participant are shown in Figure 7 as an example. Figure 7 shows the *x*-coordinate components and *y*-coordinate components of the center of each leg and of both legs, T_CUE_, T_FO1_ and T_FC1_. From the results, we confirmed that T_FO1_ and T_FC1_ can be measured in any case when the visual cues are forward, backward and sideways. Thereby, the stepping performance such as T_R1_ and T_SD1_ were obtained.

In addition, we confirmed that we can detect which square the participant stands using data of the center of each leg and of both legs. If we use only data of the center of both legs, we can not judge whether the participant moves to the exact target square.

### Test 2: statistical analysis of the test using a force platform and the measurement system simultaneously - test-retest reliability

There was high test-retest reliability of the stepping performance obtained by the measurement system (*p* < 0.001): T_R1_ (ICC_1, 1_ = 0.810; 95% confidence interval (CI), 0.485–0.951); T_SD1_ (ICC_1, 1_ = 0.831; 95% CI, 0.542–0.957).

### Test 2: statistical analysis of the test using a force platform and the measurement system simultaneously - criterion-related validity

T_R1_ and T_SD1_ determined using the developed measurement system were highly correlated with T_R1_ and T_SD1_ determined using the force platform: T_R1_ (*r* = 0.997, *p* < 0.001); T_SD1_ (*r* = 0.879, *p* < 0.001).

## Discussion

The results indicate that stepping performance was accurately and reliably measured by the developed system. There was a strong relation between reaction time and stride duration determined using the measurement system and the force platform. The magnitude of reliability was nearly equal to intraclass correlation coefficients of the stepping performance reported by Melzer et al. [16], who reported ICC of 0.91 for foot-off time and 0.76 for foot-contact time measured using a force platform. As a result, the measurement performance of the developed measurement was equivalent to that of a force platform. The measurement system has many advantages in terms of convenience, cost, and the number of parameters that can be obtained.

Compared to other step performance assessment systems, the measurement system offers several important advantages for fall prevention in communities. It is small, relatively in expensive, highly portable, and has minimal setup meaning that stepping tests can be performed quickly even if a large number of participants are targeted. These advantages make the measurement system ideal for measuring stepping in community settings. The system can obtain spatial parameters such as the stepping trajectory that is difficult to obtain by other step performance assessment systems.

Stepping performance measured by the measurement system can be used as assessment parameters [4-8]. Therefore, the system may be useful for assessing fall risk in fall prevention programs in the future. The relation between the stepping performance measured using the developed measurement system and a fall risk in elderly is under survey, and it is expected to be reported in next paper.

Dynamic balance assessments using force platforms have identified factors that are associated with fall risk and have contributed to the development of rehabilitation programs. Force platform measurements indicated that the increase in step execution time that occurs under dual-task conditions was greater in elderly fallers than in elderly non-fallers [5], and that step training improved the speed of voluntary step initiation [19]. The measurement system evaluated in the present study may enable researchers and therapists to investigate further details of balance control deficits associated with falling and verify the effects of training programs in elderly and patients at clinical sites conveniently.

## Conclusions

We tested the validity of a new measurement system that applied a laser range finder and allowed for convenient assessment of stepping performance. The measurement system is advantageous over current systems of measuring stepping performance in terms of convenience, cost, and the number of parameters that can be obtained. In this study, we applied the leg detection algorithm, and determined temporal and spatial step parameters from the position and velocity of the center of each leg and of both legs. The validity of the system to measure reaction time and stride duration was evaluated according to force platform measures, and was confirmed. This measurement system may be helpful for assessing fall risk indicators in elderly in community and clinical settings.

## Competing interests

Toshiki Moriguchi is an employee of Murata Machinery. The other authors have no competing interests.

## Authors’ contributions

TM was involved in the design and development of the measurement system, data acquisition, analysis and interpretation, and drafted the manuscript. TM was involved in the concept, design and coordination of the study, data acquisition and revision of the manuscript. MY and KU conducted the tests and were involved in experimental planning and revision of the manuscript. SN was involved in the data acquisition, analysis and interpretation and revision of the manuscript. TA was involved in the concept, design and coordination of the study and revision of the manuscript. MT was involved in the concept, design and coordination of the study, development of the measurement system, data acquisition and revision of the manuscript. All the authors have read and approved the final version of the manuscript.
